# Developing an Interprofessional Framework for Culinary Nutrition and Culinary Medicine Competencies: A Consultation with International Experts

**DOI:** 10.3390/nu18121897

**Published:** 2026-06-11

**Authors:** Emma Stirling, Olivia Thomas, Sharon Croxford

**Affiliations:** 1 Discipline of Nutrition and Dietetics, Faculty of Health Sciences, Australian Catholic University, Melbourne, VIC 3065, Australia; sharon.croxford@acu.edu.au; 2 Boston Medical Center, Boston, MA 02118, USA; olivia.thomas@bmc.org

**Keywords:** culinary nutrition (CN), culinary medicine (CM), interprofessional practice (IPP), interprofessional education (IPE), food is medicine (FIM)

## Abstract

**Background**: Despite global interest in the fast-emerging field of culinary nutrition (CN) and culinary medicine (CM), frameworks and competencies are underdeveloped. Due to the interprofessional nature and diverse research and practice applications, there is a need for a broad consideration of relevant competencies with input from experts across multiple disciplines. **Aims**: The aims of this study were to explore existing standards from related fields and create a draft framework of competency domains and elements required for CN and CM interprofessional practice and to consult with a panel of international experts in the field as a first step towards achieving consensus. **Methods**: Nine competency standards relevant to interprofessional CN and CM practice informed the mapping and development of a draft framework. A modified online Nominal Group Technique (NGT) with ten international experts was performed with facilitated discussion around the domains, elements, and guiding statements of the framework. **Results**: The resulting *Interprofessional Framework for Culinary Nutrition and Culinary Medicine Competencies* outlines a preliminary framework intended to support future refinement and validation, and acts as a prompt to consider extensive capabilities across an interprofessional team. **Conclusions**: To the authors’ knowledge, this is the first study to investigate an interprofessional framework with competency domains and elements for professionals delivering CN and CM. The framework provides an initial expert-informed foundation to support the refinement, external validation, and development of multiple specific competency standards and the evolution of future consensus regarding the delivery of CN and CM, which will be distinct to disciplines, settings, organizations, population groups, and practice areas.

## 1. Introduction

In the last ten years, there has been a growing interest in food and nutrition-related health care provided as health-related culinary interventions, designed and delivered by interprofessional teams. Despite the global interest in the fast-emerging field of culinary nutrition and culinary medicine, frameworks and competencies are underdeveloped. Due to the interprofessional nature and diverse research and practice applications, there is a need for a broad consideration of relevant competencies and expert consensus from multiple disciplines.

### 1.1. Culinary Nutrition and Culinary Medicine

Culinary nutrition is defined as the integration of culinary arts and nutrition that applies practical knowledge and skills to improve food and nutrition-related health, whereas culinary medicine is a health practitioner-led culinary nutrition intervention or activity [1]. There are many disciplines involved in CN and CM practice globally, including medical, allied health, nutrition and dietetics, culinary arts, food science, home economics, education, and paraprofessionals such as health coaching [1]. Interprofessional practice (IPP) is recommended to be the most effective approach in the design and dissemination of targeted culinary nutrition interventions, a set of activities to support nutrition and health goals and behaviour change in individuals and groups [1,2]. There is growing evidence of the positive behavioural, psychological, and health impacts of CN and CM interventions as an adjunct or alternative to traditional care [3,4,5,6,7,8,9,10,11].

### 1.2. Evolving Field of Healthcare Practice

Healthcare practice is a dynamic field marked by constant advancement, evolving through new research findings and technologies, quality improvements, shifting societal and patient needs, and the expansion of roles and professional collaboration across disciplines. Food is Medicine (FIM) is an evolving concept that encompasses a range of food-based interventions integrated into healthcare for individuals with specific medical conditions and, often, related social needs. These interventions include medically tailored meals and produce prescription programmes, with varying levels of nutrition and culinary education [12]. CN and CM are evolving from grassroots efforts and various change champions [13,14] applying conceptual derivation, borrowing concepts from other fields to fit the new [15]. CN and CM have been scaled through a process of change management to foster adoption, and in recent times, evidence-based, system-level adaptations [16]. The challenges of fast-paced change can result in implementation that precedes practice models and frameworks, which are necessary for standardization, benchmarking of a competent workforce, and consistent approaches to organizational planning. It is proposed that the development of an interprofessional competency framework, along with other emerging CN and CM frameworks [17], is a vital step to help ensure the continued growth of a high-quality, safe, effective field of CN and CM practice.

### 1.3. Competency Standard Development

**Figure 1:**
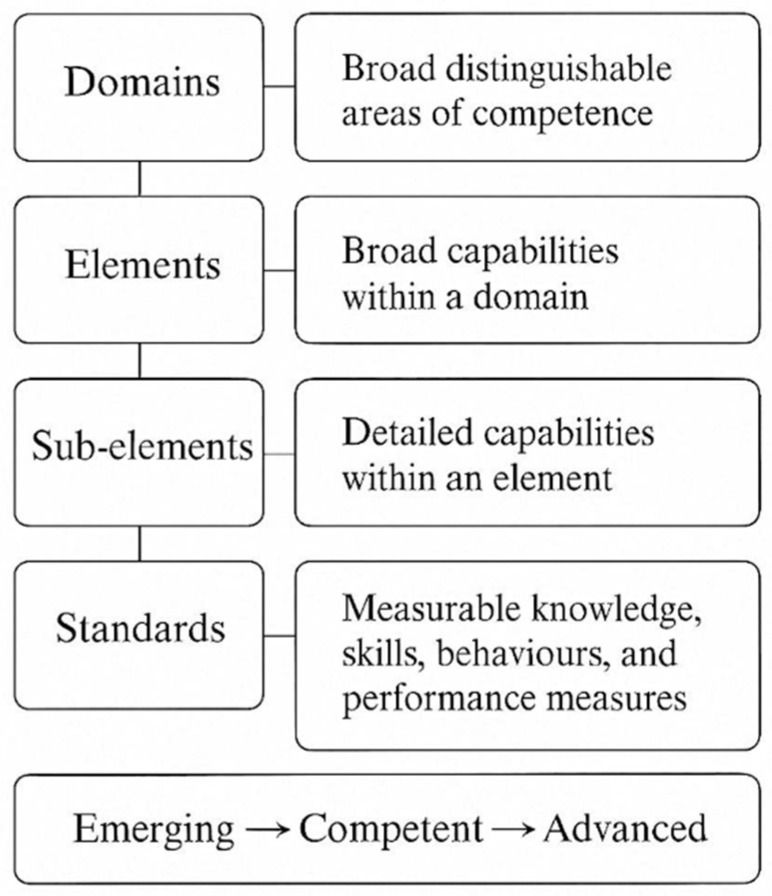
Common hierarchy observed in healthcare competency standard development.

The overarching interprofessional competency domains and elements of CN and CM have not previously been systematically explored. In healthcare, education, and related fields, multiple competency standards exist, specific to disciplines, settings, organizations, population groups, and practice areas. A common structure and hierarchy are observed in the design of competency standards in the order of domains (broad distinguishable areas) to elements (broad capabilities) to sub-elements (detailed capabilities) to competency standards (measurable knowledge, skills, and behaviours often in tiered levels of performance), as displayed in Figure 1 [18,19,20]. The use of domains and elements in this study reflects established conventions in competency framework development, where hierarchical structuring supports clarity, transferability, and application across diverse contexts.

There is often a level of consistency and overlap between domains and elements across different frameworks; however, the measurable competency standards are unique and specific. The development of an interprofessional competency framework that maps only the culinary nutrition domains and elements for interprofessional practice can support the development of multiple specific competency standards that are evolving in CN and CM and will be distinct to disciplines, settings, organizations, population groups, and practice areas [21,22]. This framework can also act as a critical prompt to consider capabilities across an interprofessional team to strengthen successful design, delivery, and impact in targeted CN and CM interventions. Development of an interprofessional competency framework through expert discussion will lead to further consistent advancements, fundamental to education, research, and practice in CN and CM.

While multiple competency standards exist across healthcare and related disciplines, and emerging frameworks in culinary nutrition and culinary medicine are beginning to be described, these are typically discipline-specific or context-bound. There remains a gap in the systematic identification and mapping of interprofessional domains and elements that underpin CN and CM practice. This study addresses this gap by proposing a high-level, interprofessional framework focused on domains and elements, designed to inform the development of multiple, context-specific competency standards across disciplines, settings, and practice areas.

### 1.4. Aims

The aims of this study were as follows:

▪ To explore existing standards from related fields and create a draft framework of competency domains and elements required for CN and CM interprofessional practice;
▪ To consult with a panel of international experts in the field as a first step towards achieving consensus.

## 2. Materials and Methods

This study reviewed existing competency standards in fields relevant to interprofessional culinary nutrition and culinary medicine practice to inform the mapping and development of a draft *Interprofessional Framework for Culinary Nutrition and Culinary Medicine Competencies*, which was subsequently refined through a modified online Nominal Group Technique with international experts. Gadamerian philosophical hermeneutics was the methodological framework for this study with ongoing reflexivity.

### 2.1. Methodological Framework

In Gadamerian philosophical hermeneutics, the concept of a fusion of horizons describes a communicative process through which understanding is developed by engaging with different perspectives, contexts, and experiences, ultimately shaping one’s own worldview [23,24]. Meaning is constructed through an ongoing, iterative consideration of individual components in relation to the whole. Interpretation, therefore, is not limited to the text itself; rather, it is influenced by the interpreter and their interaction with the world, reflecting the ontological dimension of hermeneutics. Hovey et al. [23] highlight that the complexity of human experience within social contexts and dimensions of health presents an ongoing challenge for researchers across healthcare disciplines. Each profession contributes a distinct perspective, and when these perspectives are brought together, they enable a more comprehensive understanding through a fusion of horizons.

The iterative approach of the NGT process reflects the cyclic process of Gadamer’s hermeneutic circle. The NGT, which has also been called the expert panel method, is a dominant consensus method that allows a group of experts to develop and suggest ideas/solutions within a group [25]. The NGT was applied in a virtual setting and involved the use of facilitated, structured activities within a group comprising purposefully selected, multi-disciplinary stakeholders (nominal group), with the broad aim of achieving a level of expert agreement on a draft framework [26,27].

In practical terms, the Gadamerian hermeneutic framework informed the interpretive and iterative processes underpinning this study. The concept of a fusion of horizons supported the integration of diverse disciplinary perspectives within the expert panel, while the hermeneutic circle aligned with the iterative refinement of the framework through cycles of interpretation, discussion, and revision. This approach guided both the research team’s reflexive engagement with the data and the facilitated dialogue within the Nominal Group Technique, contributing to the co-construction and refinement of the interprofessional framework.

### 2.2. Exploration of Relevant Competencies

The research team drew on domain expertise in culinary nutrition and culinary medicine, supported by the literature identifying key contributing disciplines [1,11], to identify relevant competency standards across Nutrition and Dietetics, Food Science/Technology, Culinary Arts, and Home Economics, along with competency standards on interprofessional practice. A targeted search was conducted during July 2025 using academic databases and professional peak body websites. Identified sources were reviewed, and a representative subset was selected through team consensus to inform the framework development. Inclusion criteria comprised (i) currency; (ii) English language; (iii) authorship by recognized peak bodies in the United States, United Kingdom, or Australia; and (iv) coverage across the four target disciplines, along with interprofessional practice.

Competency elements were extracted from the selected subset of standards, followed by multiple rounds of iterative mapping to inform the development of a draft framework relevant to interprofessional culinary nutrition and culinary medicine practice. Guided by established literature on competency framework development and employing a deductive analytical approach, the research team applied their expertise in competency standard design and CN and CM practice to compare and interpret the data, identifying patterns and relationships [18].

Two researchers (E.S. and S.C.) first independently extracted competency elements from each standard and clustered them based on similarities and outliers, generating two preliminary versions of the draft framework. These versions were subsequently compared and consolidated through a series of team-based discussions, involving iterative mapping, refinement of domain and element positioning, and consensus on the wording of statements. Following agreement on the domains and elements, guiding statements were developed to articulate the intent and guide dissemination. The full research team reviewed and refined the draft *Interprofessional Framework for Culinary Nutrition and Culinary Medicine Competencies* prior to progression to the NGT. A supplementary workflow and representative mapping table are provided in Supplementary Figure S1 and Supplementary Table S1. Consistent with NGT procedures, an information pack was distributed to participants in advance, including background materials and instructions to review the draft framework and prepare feedback prior to the meeting.

### 2.3. Recruitment and Sampling

Participants were recruited using purposive sampling to achieve representation across the four target disciplines. Inclusion criteria comprised (i) demonstrated expertise in CN or CM education, practice, or research; and/or (ii) recognized expertise in Nutrition and Dietetics, Food Science/Technology, Culinary Arts, or Home Economics. Purposive sampling was used to recruit participants with relevant expertise to ensure the inclusion of individuals with knowledge and experience aligned with the study’s aims. This approach allows researchers to deliberately select participants based on predefined criteria and to capture a range of perspectives (maximum variation sampling). While there is no clear consensus on the optimal size of nominal groups, previous studies have suggested that groups of approximately 8–10 participants are appropriate for effective discussion and consensus development [25]. The participants invited to the nominal group were experts practicing or researching in the main or related fields dominant in CN and CM, including nutrition and dietetics, medicine, food science, community and public health, culinary arts, and home economics. Informed consent was sought via Qualtrics using an online form with the study approved by the Human Ethics Committee of Australian Catholic University (protocol code 2025-4088E and approved on 4 February 2025). Written informed consent included permission to publish participant names with this paper.

### 2.4. Modified Nominal Group Technique

The NGT was conducted in three discrete stages: Stage 1—Individual responses; Stage 2—Clarification and consolidation of responses; Stage 3—Individual voting activities [24]. Stage 1 and Stage 2 were conducted online via a two hour Microsoft Teams meeting attended by all researchers and participants. In Stage 1, a ‘round robin’ format was performed; that is, for each domain of the draft interprofessional framework, each participant shared a single idea or recommendation, using the written chat function, and the cycle was repeated until exhausted. Participants were instructed that they could share their expert opinions on any aspect of the domain: the domain name, elements, and guiding statements. This individual approach has been recommended as a means of achieving equity in relation to participants’ contributions [25]. In Stage 2, participants were invited to seek clarification and discussion from other nominal group members using the ‘hand raising’ function. All responses were collated in real-time on an MS Excel document by one researcher (S.C.), with one week provided for further additional feedback. The meeting was recorded with consent for transcription purposes only. One researcher (S.C.) synthesized all comments and feedback on the draft interprofessional framework by grouping and merging suggestions, with subsequent iterative review and discussion by three researchers (S.C., E.S., and O.T.) to interpret, refine, and agree on modifications to the framework domains, elements, and guiding statements. This process was guided by the structured stages of the NGT and supported by iterative researcher consensus to ensure transparency and consistency in the interpretation of expert input. Three researchers (S.C., E.S., and O.T.) then discussed the proposed modified framework, item by item, considering individual researcher interpretation, and agreed to final revisions to be presented to the nominal group for Stage 3 voting. Only comments already addressed within the framework or those of a general nature, unrelated or peripheral to the draft interprofessional framework, were rationalized. The traditional NGT private voting and ranking phase was modified in Stage 3 to private voting and rating, as observed in other NGT studies [28]. The proposed revisions were presented to the nominal group as questions in an online survey via Qualtrics, with the voting option to accept, reject, or abstain for each item. A 70% threshold aggregate score was applied to form expert agreement, representing a strong majority view. This is consistent with consensus-based research practices, where agreement thresholds vary and commonly fall at approximately 70% or higher, with no universal standard established [28]. Additional qualitative data analysis, such as thematic analysis of raw data or transcripts, was not warranted in this study due to the nature of the task [29]. The researchers ensured that the NGT structure was maintained and the key stages were delivered effectively, and assisted participants in interpreting the complex information in facilitated discussion and communication.

## 3. Results

### 3.1. Developing the Draft Framework

Nine standards relevant to interprofessional CN and CM practice informed domain and element competency mapping in the first stage of this research, as outlined in Table 1 below.

**Figure 2:**
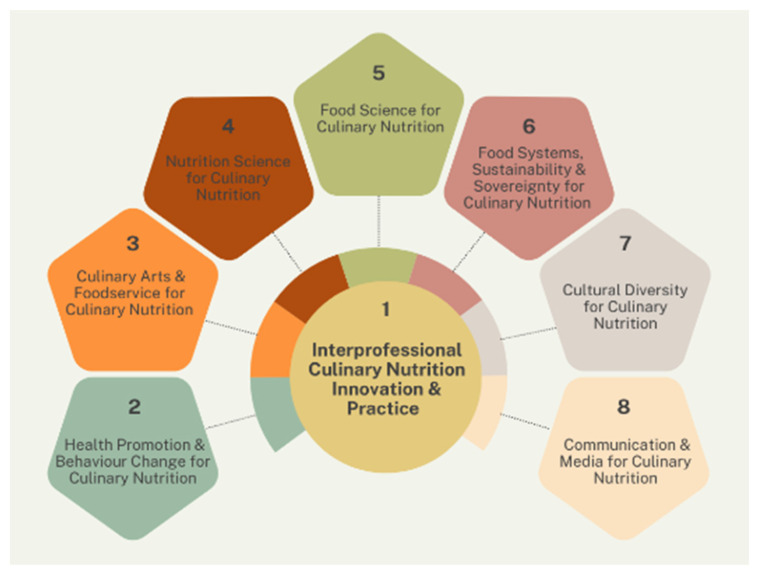
Draft infographic displaying eight domains in the draft *Interprofessional Framework for Culinary Nutrition and Culinary Medicine Competencies*.

The draft *Interprofessional Framework for Culinary Nutrition and Culinary Medicine Competencies* consisted of eight domains and multiple grouped elements (see Supplementary Material S1). The first prominent domain and elements relate to culinary nutrition interprofessional innovation and practice, with seven interconnecting domains drawing from health promotion and behaviour change; culinary arts and foodservice; nutrition science; food science; food systems, sustainability, and sovereignty; cultural diversity; and communication and media. Each element within the framework was designed to be unique, with any overlap in broad capabilities distinguished within relevant domains. For example, the term ‘sensory’ appears across several elements; however, each element is unique, such as the element ‘multi-modal sensory perception knowledge’ in Health Promotion and Behaviour Change domain; the elements ‘multi-modal sensory perception theory and practice’, ‘sensory testing of food using validated methods’, and ‘sensory evaluation’ in the Food Science domain; and the element ‘sensory gardens’ in the Food Systems, Sustainability and Sovereignty domain. Guiding statements summarized the elements within the context of each domain to assist with interpretation and future adoption and adaptation into specific competency standards. The draft *framework* included an illustrative draft infographic representing the eight domains (Figure 2), and a glossary of key terms such as ‘food is medicine’, ‘teaching kitchen’, and ‘culinary nutrition’.

### 3.2. Nominal Group—The Participants

Following sampling and recruitment, a total of 13 participants were invited, and 10 experts joined the nominal group. Experts from the USA, UK, and Australia represented the main or related identified fields dominant in CN and CM (Table 2). Two participants declined due to a lack of availability for the NGT, and one participant did not respond.

### 3.3. Nominal Group Technique and Expert Feedback

Qualitative data generated through the NGT were synthesized through iterative researcher review and discussion into key categories relating to the (i) scope and definition of the elements, (ii) structural organization and placement of elements within domains, (iii) refinement of guiding statement wording, and (iv) inclusion of additional theoretical and practice-based constructs and key terms.

Synthesized recommendations generated through the NGT process resulted in substantive refinements across all framework domains. Table 3 displays the NGT synthesized recommendations, voting outcomes, and resulting framework refinements across domains, while Table 4 provides a detailed example of the iterative modifications and restructuring undertaken within Domain 2: Culinary Nutrition: Health Promotion and Behaviour Change. In the Domain 2 example, the red text shows that the wording around theories, models, and frameworks of behaviour change was refined to avoid the promotion of specific tools, and *cooking* was added to mindful eating, reflecting culinary nutrition approaches to mindfulness techniques. Collectively, these refinements strengthened the theoretical integration and interprofessional applicability of the framework.

**Figure 3:**
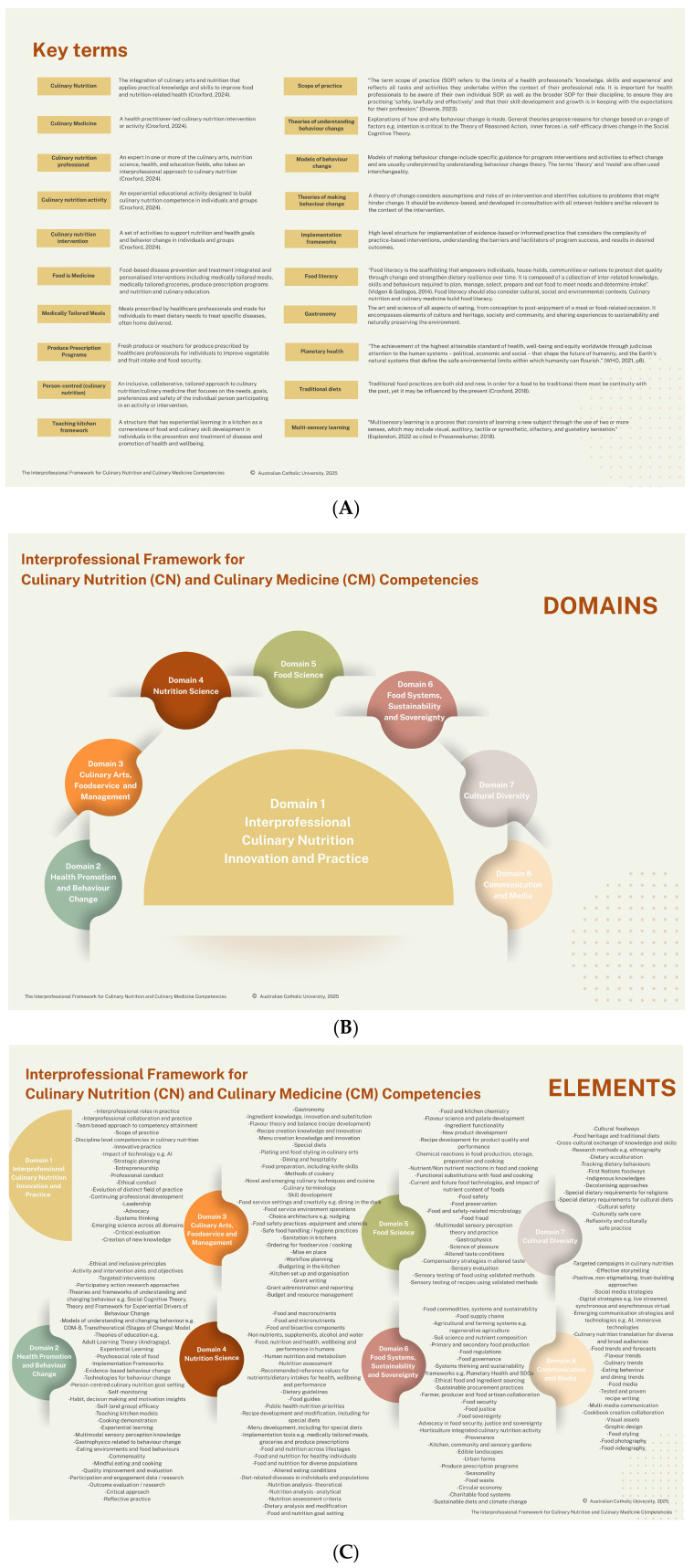

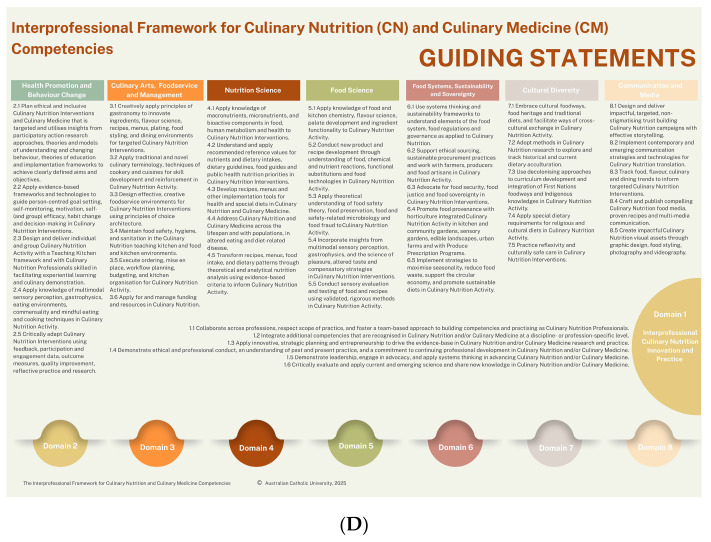
Final figures in the *Interprofessional Framework for Culinary Nutrition and Culinary Medicine Competencies* displaying (**A**) key terms [1,42,43,44,45,46], (**B**) domains, (**C**) elements, and (**D**) guiding statements. The *Interprofessional* *Framework for Culinary Nutrition and Culinary Medicine Competencies* is available as a full resource PDF download https://tinyurl.com/4nvzvj2b (accessed on 15 July 2025) [41].

In stage 3 voting, the research team presented 35 synthesized recommendations to all 10 participants via the Qualtrics online platform, with all participants conducting independent voting within 4 weeks of the NGT virtual meeting. A 70% aggregate score was applied to accept or reject the recommendations, with all 35 recommendations meeting the threshold for acceptance, forming expert agreement on the final *Interprofessional Framework for Culinary Nutrition and Culinary Medicine Competencies* (Figure 3). The *framework* provides a summary of key terms (A), then in (B), outlines the domains (broad distinguishable areas) presented graphically as if coming together to dine at the same table. Next (C), the elements (broad capabilities within a Domain) are presented as unique elements, with each phrased as a stand-alone statement within the respective domain. Finally, the *framework* summarizes the domains and elements into (D) guiding statements to help with the interpretation of the resource and provide guidance on the future development and consensus of multiple, specific sub-elements and competency standards or complementary frameworks. The *Interprofessional* *Framework for Culinary Nutrition and Culinary Medicine Competencies* is also available as a full resource PDF download [41].

## 4. Discussion

To the authors’ knowledge, this is the first study investigating an interprofessional competency framework with domains (broad, distinguishable areas) and elements (broad capabilities) for CN and CM practice. Findings demonstrate that the nominal group technique is a valuable approach to seek expert discussion and agreement in a facilitated format, particularly in an emerging, dynamic, global field. The resulting *Interprofessional Framework for Culinary Nutrition and Culinary Medicine Competencies* [41] includes unique elements grouped into domains and summarized into guiding statements, to act as a high-level, broad map to inform development of multiple, specific competency standards and future consensus that may support critical consistency in education, research, and practice in CN and CM.

The *Interprofessional Framework for Culinary Nutrition and Culinary Medicine Competencies* [41] is intended to act as a critical prompt and the architecture for a comprehensive consideration of the domains (broad distinguishable areas) and elements (broad capabilities) when building a CN or CM team, with this interprofessional approach already evident in existing programmes and research protocols [5,6]. A key benefit of IPP is that professionals share their unique expertise to create a synergistic effect, leading to better overall solutions [47,48] and high-quality care [49]. The elements required for success in CN and CM are unlikely to be achieved by individuals, and it is recommended that IPP continue to drive high-performing teams designing and delivering CN and CM interventions with impact. Multi-disciplinary members will bring distinct capabilities to collectively create an interprofessional entity, with the future likely evolving towards consensus on new, specific interprofessional competency standards assessed against a collective ensemble. All members of the team will require IPP capabilities as outlined in Domain 1 Elements within the *framework* to respect the scope of practice and optimize collaboration.

The *Interprofessional Framework for Culinary Nutrition and Culinary Medicine Competencies* [41] may help inform future consensus-building and ongoing refinement of multiple specific competency standards evolving in CN and CM distinct to disciplines, settings, organizations, population groups, and practice areas. It is vital that disciplines and professions think unconventionally and explore the full breadth of capabilities important to CN and CM when developing their respective, specific competency standards. There is work underway in the development of Nutrition Competencies for Medical Students and Physician Trainees in response to growing interest in CM [50]. The existing competencies of Registered Dietitian Nutritionists (CRDNs), which prepare RDNs for integration within Food is Medicine, have recently been investigated [51,52]. Advocacy to include further culinary competencies has been observed in dietetics [53], with the Academy of Nutrition and Dietetics Food and Culinary Professionals Dietetic Practice Group (FCP DPG) previously developing core food and culinary competencies [30]. There is also evidence of integration of nutrition competencies into disciplines associated with the culinary arts. The American Culinary Federation (ACF) Accreditation Standards and Required Knowledge and Skill Competencies for Culinary Programs include a domain on nutrition [34], and de Thomas et al., [54] conducted a review into CN in the gastronomic sciences. Along with professional competency standards and consensus, the future will also see the development of multiple, specific patient, client, and participant competency frameworks and standards [22].

The *Interprofessional Framework for Culinary Nutrition and Culinary Medicine Competencies* [41] may also act as a mapping tool in the design of innovative curricula and programmes attracting future students from diverse areas of interest to the field of CN and CM. To meet training demands for the workforce, an increase in CN and CM education and continuing professional development offerings has been observed, from short courses to dedicated postgraduate tertiary qualifications. Examples of discipline-specific programmes at the tertiary level include The Nourish Program at the University of Texas Health Science Center at Houston School of Public Health to train Registered Dietitian Nutritionists (RDNs) in culinary medicine [55,56], the University of Texas Southwestern’s Culinary Medicine Program [57], and the University of Vermont Culinary Medicine Program, including an elective for medical students [58]. Examples of interprofessional education [IPE] programmes are the Harvard Medical School short course, Culinary Health Education Fundamentals (CHEF) Coaching [59]; Tulane University and The American College of Culinary Medicine’s Certified Culinary Medicine Specialist (CCMS) [60]; and Australian Catholic University’s tertiary qualification, the Graduate Certificate in Culinary Nutrition Science, along with micro credentials and short courses [61]. Investigating training needs, Asher et al. [62] found that Australian health and education professionals are interested in continuing professional development to enhance their knowledge of CN education and behaviour change support. Brennan et al. [63] found that a CM course for medical students and health science graduates at the University of Utah provided contextual learning experiences that enhanced participants’ confidence in counselling patients on diet and lifestyle, with potential benefits for team-based clinical care. Researchers and leadership organizations, such as the Teaching Kitchen Collaborative, are advocating for global consistencies and tools such as this *framework* may help inform future consensus of universal pedagogy and credentialing of providers in CN and CM education [16,22].

While this research provides a critical first step in describing interprofessional practice in CN and CM, and was developed from relevant existing competency frameworks and descriptions, the researchers acknowledge that both their own and the expert nominal group panel’s credentials and experiences likely introduced some bias to the research process. While illustrative examples of existing programmes highlight the relevance and potential application of the *framework*, formal validation and empirical testing were beyond the scope of this study and represent important directions for future research. The findings are based on agreement within the expert panel and have not yet been tested or evaluated beyond this group. This limitation is balanced by the recruitment of experts from across the globe from key professional practice areas, and the ongoing discussion between researchers at each of the key stages of the research to present an objective framework. The researchers also acknowledge that the *Interprofessional Framework for Culinary Nutrition and Culinary Medicine Competencies* [41] only maps the domains and elements and is not a comprehensive interprofessional competency standard. Future work is also required with broader stakeholder involvement and comprehensive iterative refinement to achieve consensus on competency frameworks within CN and CM practice.

## 5. Conclusions

The *Interprofessional Framework for Culinary Nutrition and Culinary Medicine Competencies* [41] developed here represents an initial expert-informed first step to map the domains (broad distinguishable areas) and elements (broad capabilities) for interprofessional practice in CN and CM. This work can be likened to an important, early exploratory map intended to guide ongoing refinement, validation, and future consensus development. It provides a global, interprofessional view to inform further discoveries and the charting of rich detail and navigation ahead in emerging CN and CM competencies. Future directions may include national and international organizations, professional and peak bodies, and other researchers proposing additional or alternate competency frameworks; the development of multiple, specific CN and CM competency standards distinct to disciplines, settings, organizations, population groups, and practice areas; and advances in competency assessment, including interprofessional team collective assessment, programmatic approaches, or entrustable professional activities [64]. Continued international collaboration, broader stakeholder engagement, and external validation studies will be important to further refine and establish consensus around competency standards in CN and CM.

## Figures and Tables

**Table 1:** Standards with competency domains and elements included in the draft foundational framework mapping, grouped by field.

Organization: Title	Field
Academy of Nutrition and Dietetics: Food and Culinary Professionals Dietetic Practice Group: FCP Core Competencies [30]	Nutrition Dietetics
Nutrition Society of Australia: Nutrition Science Core Competencies for Undergraduate Nutrition Degrees [31]	Nutrition
Association for Nutrition: Core Competency Requirements for Registered Nutritionists [32]	Nutrition
Society for Nutrition Education and Behavior: Nutrition Education Competencies [33]	Nutrition Education
American Culinary Federation (ACF) Accreditation Standards and Required Knowledge and Skill Competencies for Culinary Programs [34]	Culinary arts
Research Chefs Association Core Competencies for the Practicing Culinologist [35,36]	Culinary arts Food technology
Institute for Food Technologists (IFT) Education Standards in Food Science [37]	Food technology
International Federation for Home Economics: Home Economics in the 21st Century Position Statement [38,39]	Home economics
Interprofessional Education Collaborative: IPEC Core Competencies for Interprofessional Collaborative Practice [40]	Interprofessional healthcare practice

**Table 2:** Nominal group expert participants in alphabetical order by surname.

Name	Position(s)	Company/ Institution, Country	Relevant Field(s)
Associate Professor Jaclyn Albin, MD	Inaugural Director, UTSW’s Culinary Medicine Program	University of Texas Southwestern Medical Centre, USA	Medicine; Public Health; Education
Deanne Brandstetter MBA, RD	Director	Culinary Nutrition Strategy LLC, USA	Foodservice; Nutrition & Dietetics
Ghislaine Challamel	Senior Advisor	Teaching Kitchen Collaborative, USA	Multi-disciplinary teaching kitchens; Food science
Lynn Fredericks	Founder	FamilyCook Productions, USA	Community-based culinary nutrition
Benjamin M. T. Gill APD	Lecturer in Culinary Nutrition	Australian Catholic University, Australia	Food science; Nutrition & Dietetics; Education
Dr Fiona Lavelle	Lecturer in Nutritional Sciences	King’s College London, UK	Nutrition & Dietetics; Behavioural Sciences; Education
Dr Jennifer Massa	Lead Research Scientist, Nutrition	Harvard T.H. Chan School of Public Health, USA	Nutrition; Research
Professor Donna Pendergast	Director Engagement	Griffith University, Australia	Home Economics; Education
R. Leah Pryor	Culinary Medicine Manager, Chef Educator Culinary Chair	University of Vermont, USA Teaching Kitchen Collaborative, USA	Culinary Arts; Education
Milette Siler RD	Culinary Dietitian and Co-Founder	Culinary Medicine, University of Texas Southwestern, USA	Nutrition & Dietetics Education

**Table 3:** Synthesized Nominal Group Technique (NGT) recommendations, voting outcomes (70% consensus threshold), and resulting framework refinements.

NGT Survey Item/Proposed Modification	Voting Outcome	Subsequent Framework Refinement
Domain 1: Interprofessional Culinary Nutrition Innovation and Practice		
Add/edit statement 1.3 to include “Culinary Nutrition and/or Culinary Medicine” terminology within innovation, strategic planning, and entrepreneurship concepts	8/8 accepted (100%); 2 abstentions	Refined terminology to strengthen integration of Culinary Nutrition and Culinary Medicine concepts within innovation and evidence-generation statements
Add/edit statement 1.4 to strengthen ethical and professional conduct and continuing professional development concepts	10/10 accepted (100%)	Expanded ethical conduct and continuing professional development concepts across the framework
Add/edit statement 1.5 to strengthen leadership, advocacy, and systems-thinking concepts,	10/10 accepted (100%)	Strengthened leadership, advocacy, and systems-thinking language within interprofessional practice statements
Add/edit elements and statement 1.3 to include innovation, strategic planning, entrepreneurship, and impact of technology, including AI	8/10 accepted (80%); 2 rejections	Added innovation, technology, and emerging practice concepts, including AI-related considerations
Add/edit elements and statement 1.1 to strengthen interprofessional roles in practice, collaboration, team-based competency attainment, and scope of practice	10/10 accepted (100%)	Expanded interprofessional collaboration and scope-of-practice concepts within competency attainment and practice statements
Add/edit elements and statement 1.4 to include evolution of a distinct field of practice	8/9 accepted (88.9%); 1 rejection and 1 abstention	Refined professional identity and evolving field-of-practice concepts
Create new statement 1.6 and associated elements relating to emerging science, critical evaluation, and creation of new knowledge	10/10 accepted (100%)	Added concepts relating to emerging science, critical evaluation, and evidence generation in Culinary Nutrition and Culinary Medicine
Domain 2: Health Promotion and Behaviour Change		
Reorder statements by moving original 2.4 to become new 2.2	10/10 accepted (100%)	Reordered framework structure to improve conceptual flow of behaviour change concepts
Move behaviour change theories element into 2.1 and add psychosocial role of food	9/9 accepted (100%); 1 abstention	Consolidated behaviour change theory concepts and integrated psychosocial role of food within intervention planning
Add theories and frameworks of understanding and changing behaviour, theories of education, and participatory action research to 2.1	10/10 accepted (100%)	Expanded theoretical and educational frameworks underpinning behaviour change interventions
Add Food is Medicine implementation frameworks to 2.1	10/10 accepted (100%)	Strengthened implementation science and Food is Medicine concepts
Add self-(and group) efficacy to new 2.2	10/10 accepted (100%)	Expanded self-efficacy and group efficacy concepts within behaviour change practice
Add mindful eating and cooking techniques to new 2.4	10/10 accepted (100%)	Expanded mindful eating and mindful cooking concepts within sensory and behavioural practice
Domain 3: Culinary Arts, Foodservice and Management		
Rename Domain 3 to “Culinary Arts, Food Service and Management”	10/10 accepted (100%)	Refined domain terminology relating to foodservice and management
Add creativity terminology to statements 3.1 and 3.2	9/10 accepted (90%); 1 rejection	Strengthened creativity and innovation language within culinary practice statements
Add “ingredient knowledge, innovation, and substitution” to 3.1 elements	10/10 accepted (100%)	Expanded ingredient innovation and substitution concepts
Refine flavour theory and balance elements to distinguish recipe development concepts	9/10 accepted (90%)	Clarified flavour science and recipe development terminology
Add “skill development” to 3.2 elements and statement	10/10 accepted (100%)	Expanded culinary skill development and reinforcement concepts
Create new 3.6 relating to grant writing, grant administration, and resource management	8/9 accepted (88.9%); 1 rejection and 1 abstention	Added funding acquisition, grant administration, and resource management concepts
Domain 4: Nutrition Science		
Add “supplements” to 4.1 elements	9/9 accepted (100%); 1 abstention	Expanded terminology relating to non-nutrients and supplements
Add implementation tools, including medically tailored meals, groceries, and produce prescriptions to 4.3	10/10 accepted (100%)	Strengthened implementation and Food is Medicine terminology
Revise lifespan food and nutrition terminology in 4.4	10/10 accepted (100%)	Expanded lifespan nutrition terminology
Revise 4.5 statement from “Evaluate” to “Transform”	8/10 accepted (80%); 2 rejections	Refined terminology relating to nutrition analysis and modification concepts
Domain 5: Food Science		
Add flavour science and palate development concepts to 5.1	8/9 accepted (88.9%); 1 rejection and 1 abstention	Expanded flavour science and palate development concepts
Add food fraud to 5.3 elements and statement	8/9 accepted (88.9%); 1 rejection and 1 abstention	Expanded food safety, microbiology, and food fraud concepts
Domain 6: Food Systems, Sustainability, and Sovereignty		
Add regenerative agriculture, soil science, and sustainability frameworks to 6.1	10/10 accepted (100%)	Expanded sustainability science, regenerative agriculture, and systems-thinking concepts
Collapse separate garden elements into “kitchen, community, and sensory gardens”	9/10 accepted (90%)	Streamlined horticulture and garden-related concepts
Add seasonality and charitable food systems to 6.5	10/10 accepted (100%)	Expanded sustainability, seasonality, and charitable food systems concepts
Domain 7: Cultural Diversity		
Add food heritage and cross-cultural exchange concepts to 7.1 and move psychosocial role of food to Domain 2	10/10 accepted (100%)	Expanded cultural food heritage and cross-cultural exchange concepts, and repositioned psychosocial role of food within behaviour change
Add decolonising approaches to 7.3	10/10 accepted (100%)	Strengthened decolonising approaches, First Nations foodways, and Indigenous knowledges concepts
Domain 8: Communication and Media		
Add targeted, non-stigmatizing, and trust-building approaches to 8.1	10/10 accepted (100%)	Expanded communication and audience engagement approaches
Add digital, virtual, AI, and translation strategies to 8.2	7/9 accepted (77.8%); 2 rejections and 1 abstention	Expanded emerging communication technologies and translation concepts
Add food trends and forecasts to 8.3	9/9 accepted (100%); 1 abstention	Expanded trend forecasting concepts within culinary nutrition communication
Add cookbook creation collaboration to 8.4	9/9 accepted (100%); 1 abstention	Expanded multimedia communication and publishing concepts
Glossary and Terminology		
Add glossary terms, including theories/frameworks of change, teaching kitchen framework, implementation frameworks, food literacy, gastronomy, Planetary Health, traditional diets, multisensory learning, scope of practice, and person-centred culinary nutrition	10/10 accepted (100%)	Expanded glossary definitions and supporting terminology to improve conceptual clarity and interpretability across disciplines and settings
Rename Domains 3–5 to include “science” terminology aligned with definition of Culinary Nutrition Science [1]	9/10 accepted (90%)	Refined domain terminology to align with Culinary Nutrition Science conceptual definitions

**Table 4:** **Example of synthesized modifications to Domain 2 draft (A) and final (B) following the NGT process**.

(**A**)	
**Draft Domain 2 Culinary Nutrition: Health Promotion and Behaviour Change**	
**Elements**	**Guiding statements**
Ethical and inclusive principles	Plan ethical and inclusive **Culinary Nutrition Interventions** and **Culinary Medicine** that is targeted and utilizes insights from participatory action research, models of behaviour change, and **Food is Medicine** frameworks to achieve clearly defined aims and objectives
Activity and intervention aims and objectives	
Targeted interventions	
Participatory action research	
Models of behaviour change	
Food is Medicine Frameworks	
Teaching kitchens models	Design and deliver individual and group **Culinary Nutrition Activities** with a Teaching Kitchen framework and with **Culinary Nutrition Professionals** skilled in facilitating experiential learning and culinary demonstration
Cooking demonstration	
Experiential learning	
Multimodal sensory perception knowledge	Apply knowledge of multimodal sensory perception, gastrophysics, eating environments, commensality, and mindful eating techniques in **Culinary Nutrition Activity**
Gastrophysics related to behaviour change	
Eating environments and food behaviours	
Commensality	
Mindful eating	
Behaviour change theories	Apply behavioural theories, evidence-based frameworks, and technologies to guide person-centred goal setting, self-monitoring, motivation, habit change, and decision-making in **Culinary Nutrition Interventions**
Evidence-based behaviour change	
Technologies to change behaviour	
Person-centred culinary nutrition goal setting	
Self-monitoring	
Habit, decision-making, and motivation insights	
Quality improvement and evaluation	Critically adapt **Culinary Nutrition Interventions** using feedback, participation, and engagement data, outcome measures, quality improvement, reflective practice, and research
Participation and engagement data/research	
Outcome evaluation/research	
Critical approach	
Reflective practice	
(**B**)	
**Final Domain 2 Culinary Nutrition: Health Promotion and Behaviour Change**	
**Elements**	**Guiding statements**
Ethical and inclusive principles	2.1 Plan ethical and inclusive **Culinary Nutrition Interventions** and **Culinary Medicine** that is targeted and utilises insights from participatory action research **approaches**, theories and models of understanding and changing behaviour change, theories of education and **Food is Medicin****e** implementation frameworks to achieve clearly defined aims and objectives
Activity and intervention aims and objectives	
Targeted interventions	
Participatory action research approaches	
Theories and frameworks of understanding and changing behaviour change e.g.	
Models of understanding and changing behaviour change e.g.	
Theories of education e.g.	
Psychosocial role of food	
Food is Medicine Implementation frameworks	
Behaviour change theories	2.2 Apply behavioural theories, evidence-based frameworks and technologies to guide person-centred goal setting, self-monitoring, motivation, self-(and group) efficacy, habit change and decision-making in **Culinary Nutrition Interventions**
Evidence-based behaviour change	
Technologies for change behaviour	
Person-centred culinary nutrition goal setting	
Self-monitoring	
Habit, decision-making, and motivation insights	
Self-(and group) efficacy	
Teaching kitchens models	2.3 Design and deliver individual and group **Culinary Nutrition Activity** with a Teaching Kitchen framework and with **Culinary Nutrition Professionals** skilled in facilitating experiential learning and culinary demonstration
Cooking demonstration	
Experiential learning	
Multimodal sensory perception knowledge	2.4 Apply knowledge of multimodal sensory perception, gastrophysics, eating environments, commensality, and mindful eating and cooking techniques in **Culinary Nutrition Activity**
Gastrophysics related to behaviour change	
Eating environments and food behaviours	
Commensality	
Mindful eating and cooking	
Quality improvement and evaluation	2.5 Critically adapt **Culinary Nutrition Interventions** using feedback, participation, and engagement data, outcome measures, quality improvement, reflective practice, and research
Participation and engagement data/research	
Outcome evaluation/research	
Critical approach	
Reflective practice	

## Data Availability

The data presented in this study are available in the Supplementary Materials and on request from the corresponding author.

## Supplementary Materials

The following supporting information can be downloaded at https://www.mdpi.com/article/10.3390/nu18121897/s1, Supplementary Figure S1: Framework development and iterative mapping workflow; Supplementary Table S1: Representative mapping of competency standards informing framework development; Supplementary Material S1: Draft Interprofessional Framework for Culinary Nutrition and Culinary Medicine Competencies used during NGT refinement.

## Abbreviations

The following abbreviations are used in this manuscript:

CN	Culinary Nutrition
CM	Culinary Medicine
NGT	Nominal Group Technique
FIM	Food is Medicine
