# Supplementary material for: Developing an Interprofessional Framework for Culinary Nutrition and Culinary Medicine Competencies: A Consultation with International Experts
Source: Nutrients. 2026 Jun 11;18(12):1897. doi: 10.3390/nu18121897 (PMC13305353; doi:10.3390/nu18121897)
Supplement: Supplementary file 1 [file nutrients-18-01897-s001.zip › nutrients-4265527-supplementary.pdf]

Supplementary Materials

Developing an Interprofessional Framework for Culinary Nutrition and Culinary Medicine Competencies

These supplementary materials provide additional methodological detail, framework mapping transparency, and high-resolution framework figures supporting development of the Interprofessional Framework for Culinary Nutrition (CN) and Culinary Medicine (CM) Competencies.

Framework development involved iterative and interpretive mapping of competency concepts extracted from nine representative standards across Nutrition and Dietetics, Food Science/Technology, Culinary Arts, Home Economics, and Interprofessional Practice. Multiple standards informed overlapping domains and elements through independent extraction, clustering, iterative refinement, and team consensus processes.

- **Supplementary Figure S1.** Framework development and iterative mapping workflow.
- **Supplementary Table S1.** Representative mapping of competency standards informing framework development.
- **Supplementary Material S1.** Draft Interprofessional Framework for Culinary Nutrition and Culinary Medicine Competencies used during NGT refinement.

Figure S1. Framework development and iterative mapping workflow

Workflow illustrating iterative extraction, clustering, mapping, refinement, and expert consensus procedures used in framework development.

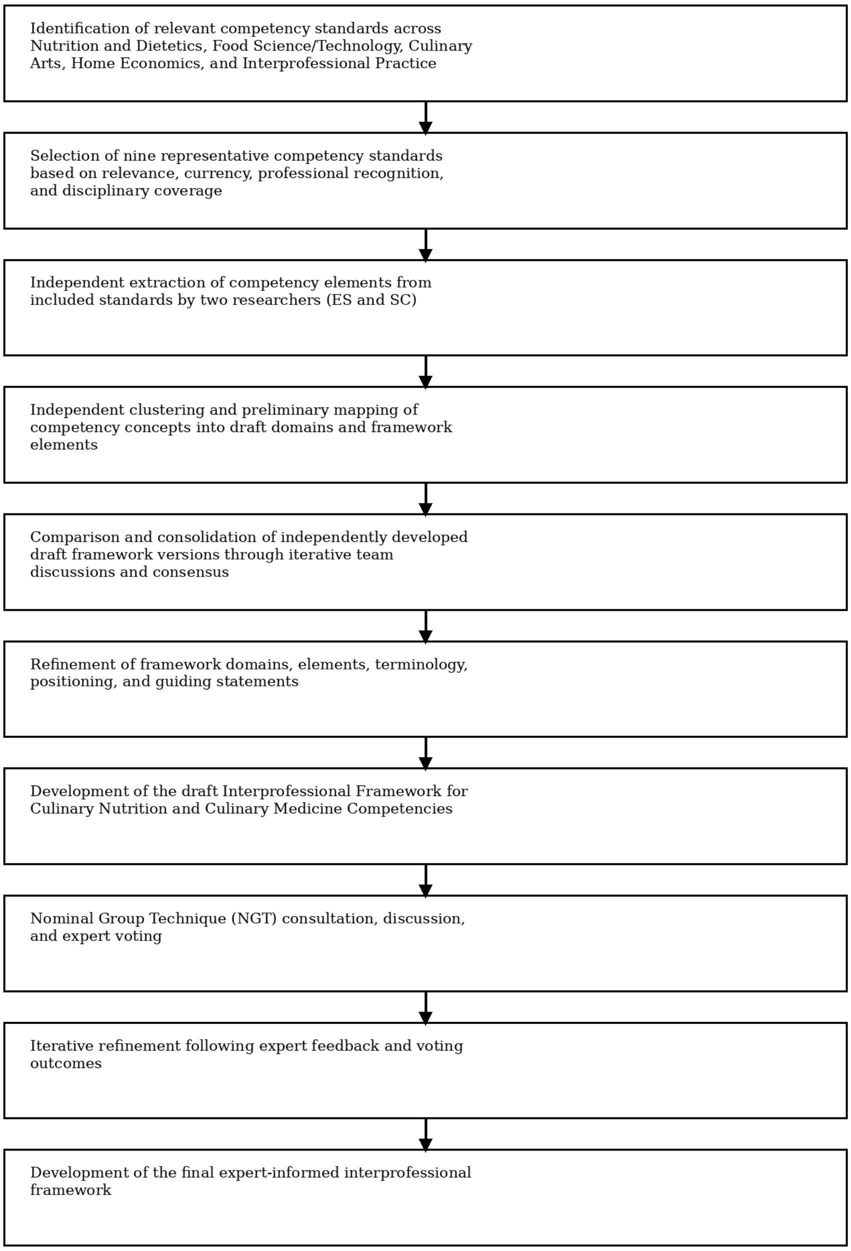

Supplementary Table S1. Representative mapping of competency standards informing framework development

Competency Standard	Representative Competency Areas/Concepts Extracted	Example Resulting Framework Domains/Elements
Academy of Nutrition and Dietetics – Food and Culinary Professionals Core Competencies	Culinary skills, menu planning, food safety, nutrition application	Domain 3: Culinary Nutrition Science: Culinary Arts, Foodservice and Management; Domain 4: Culinary Nutrition Science: Nutrition Science
Nutrition Society of Australia – Nutrition Science Core Competencies	Nutrition science, evidence-based practice, health promotion	Domain 4: Culinary Nutrition Science: Nutrition Science; Domain 2: Culinary Nutrition: Health Promotion and Behaviour Change
Association for Nutrition – Core Competencies for Registered Nutritionists	Communication, food systems, professional practice	Domain 8: Culinary Nutrition: Communication and Media; Domain 6: Culinary Nutrition: Food Systems, Sustainability and Sovereignty
Society for Nutrition Education and Behavior Competencies	Behaviour change, nutrition education, community engagement	Domain 2: Culinary Nutrition: Health Promotion and Behaviour Change
American Culinary Federation Competencies	Culinary techniques, gastronomy, foodservice operations	Domain 3: Culinary Nutrition Science: Culinary Arts, Foodservice and Management
Research Chefs Association Competencies	Product development, sensory science, food innovation	Domain 5: Culinary Nutrition Science: Food Science; Domain 3: Culinary Nutrition Science: Culinary Arts, Foodservice and Management
Institute for Food Technologists Competencies	Food safety, microbiology, sensory evaluation, food processing	Domain 5: Culinary Nutrition Science: Food Science
International Federation for Home Economics Position Statement	Food literacy, sustainability, household food practices, culture	Domain 6: Culinary Nutrition: Food Systems, Sustainability and Sovereignty; Domain 7: Culinary Nutrition: Cultural Diversity
Interprofessional Education Collaborative (IPEC) Competencies	Team-based care, interprofessional communication, role clarity	Domain 1: Interprofessional Culinary Nutrition Innovation and Practice

Supplementary Material S1. Draft Interprofessional Framework used during NGT refinement

Domain	Element	Domain and element narrative statements
**Domain 1: Interprofessional Culinary Nutrition Innovation and Practice**		
Domain 1: Interprofessional Culinary Nutrition Innovation and Practice	Interprofessional collaboration and practice	Collaborate across professions, respect scope of practice, and foster a team-based approach to building competencies with **Culinary Nutrition Professionals**.
	Team based approach to competency attainment	
	Scope of practice	
	Discipline level competencies in culinary nutrition	Integrate additional competencies that are recognised in **Culinary Nutrition** or **Culinary Medicine** at a discipline- or profession-specific level
	Innovative practice	Apply innovative, strategic planning and entrepreneurship to drive the evidence-base in **Culinary Nutrition** and **Culinary Medicine** research and practice
	Strategic planning	
	Entrepreneurship	
	Professional conduct	Demonstrate ethical and professional conduct, and commitment to continuing professional development in **Culinary Nutrition** and **Culinary Medicine**
	Ethical conduct	
	Continuing professional development	
	Leadership	Demonstrate leadership, engage in advocacy, and apply systems thinking in advancing **Culinary Nutrition** and **Culinary Medicine**
	Advocacy	
	Systems thinking	
**Domain 2: Culinary Nutrition: Health Promotion and Behaviour Change**		
Domain 2: Culinary Nutrition: Health Promotion and Behaviour Change	Ethical and inclusive principles	Plan ethical and inclusive **Culinary Nutrition Interventions** and **Culinary Medicine** that is targeted and utilises insights from participatory action research, models of behaviour change and **Food is Medicine** frameworks to achieve clearly defined aims and objectives
	Activity and intervention aims and objectives	
	Targeted interventions	
	Participatory action research	
	Models of behaviour change	
	Food is Medicine Frameworks	
	Teaching kitchens models	Design and deliver individual and group **Culinary Nutrition Activity** with a Teaching Kitchen framework and with **Culinary Nutrition Professionals** skilled in facilitating experiential learning and culinary demonstration
	Cooking demonstration	
	Experiential learning	
	Multimodal sensory perception knowledge	Apply knowledge of multimodal sensory perception, gastrophysics, eating environments, commensality and mindful eating techniques in **Culinary Nutrition Activity**
	Gastrophysics related to behaviour change	
	Eating environments and food behaviours	
	Commensality	
	Mindful eating	
	Behaviour change theories	Apply behavioural theories, evidence-based frameworks and technologies to guide person-centred goal setting, self-monitoring, motivation, habit change and decision-making in **Culinary Nutrition Interventions**
	Evidence-base behaviour change	
	Technologies to change behaviour	
	Person-centred culinary nutrition goal setting	
	Self-monitoring	
	Habit, decision making and motivation insights	
	Quality improvement and evaluation	Critically adapt **Culinary Nutrition Interventions** using feedback, participation and engagement data, outcome measures, quality improvement, reflective practice and research
	Partipication and engagement data / research	
	Outcome evaluation / research	
	Critical approach	
	Reflective practice	

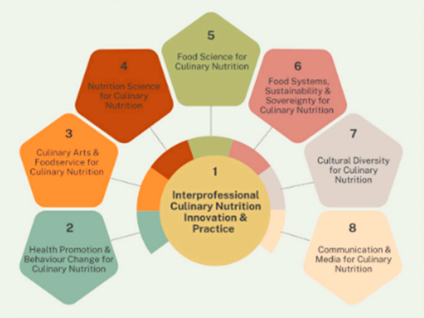

Glossary

**Culinary Nutrition**	The integration of culinary arts and nutrition that applies practical knowledge and skills to improve food and nutrition-related health.
**Culinary Medicine**	A health practitioner-led culinary nutrition intervention or activity.
**Culinary Nutrition Professional**	An expert in one or more of the culinary arts, nutrition science, health, and education fields, who takes an interprofessional approach to culinary nutrition.
**Culinary Nutrition Activity**	An experiential educational activity designed to build culinary nutrition competence in individuals and groups.
**Culinary Nutrition Intervention**	A set of activities to support nutrition and health goals and behavior change in individuals and groups.
**Food is Medicine**	A framework that integrates food-based nutrition strategies into healthcare systems and population health to address specific health needs. This approach includes interventions such as medically tailored meals, medically tailored groceries, and produce prescription programs, aiming to improve nutrition security and health outcomes.
**Medically Tailored Meals (MTMs)**	Customised meals designed for patients with specific health conditions.
**Produce Prescription Programs**	Initiatives where healthcare providers prescribe fruits and vegetables to patients, often redeemable at local markets.

42		[illegible]	
43	**Domain 3: Culinary Nutrition: Culinary Arts and Foodservice**		
44	Domain 3: Culinary Nutrition: Culinary Arts and Foodservice	Gastronomy	Apply principles of gastronomy to innovate ingredients, flavour science, recipes, menus, plating, food styling, and dining environments for targeted **Culinary Nutrition Interventions**
45		Ingredient knowledge and innovation	
46		Flavour theory and balance	
47		Recipe creation knowledge and innovation	
48		Menu creation knowledge and innovation	
49		Special diets	
50		Plating and food styling in culinary arts	
51		Dining and hospitality	
52		Food preparation, including knife skills	Apply traditional and novel culinary terminology, techniques of cookery and cuisines in **Culinary Nutrition Activity**
53		Methods of cookery	
54		Novel and emerging culinary techniques and cuisine	
55		Culinary terminology	
56		Food service settings and creativity e.g. dining in the dark	Design effective, creative foodservice environments for **Culinary Nutrition Interventions** using principles of choice architecture
57		Food service environment operations	
58		Choice architecture e.g. nudging	
59		Food safety practices	Maintain food safety, hygiene, and sanitation in the **Culinary Nutrition** teaching kitchen and food and kitchen environments
60		Safe food handling / hygiene practices	
61		Sanitation in kitchens	
62		Ordering for foodservice / cooking	Execute ordering, mise en place, workflow planning, budgeting, and kitchen organisation for **Culinary Nutrition Activity**
63		Mise en place	
64		Workflow planning	
65		Budgeting in the kitchen	
66		Kitchen set up and organisation	
67	**Domain 4: Culinary Nutrition: Nutrition Science**		
68	Domain 4: Culinary Nutrition: Nutrition Science	Food and macronutrients	Apply knowledge of macronutrients, micronutrients, and bioactive components in food, human metabolism and health to **Culinary Nutrition Interventions**
69		Food and micronutrients	
70		Food and bioactive components	
71		Non nutrients, alcohol and water	
72		Food, nutrition and health, wellbeing and performance in humans	
73		Human nutrition and metabolism	
74		Nutrition assessment	Understand and apply recommended reference values for nutrients and dietary intakes, dietary guidelines, food guides and public health nutrition priorities in **Culinary Nutrition Interventions**
75		Recommended reference values for nutrients/dietary intakes for health, wellbeing and performance	
76		Dietary guidelines	
77		Food guides	
78		Public health nutrition priorities	
79		Recipe development and modification, including for special diets	Develop recipes, menus and **Medically Tailored Meals** for health and special diets in **Culinary Nutrition** and **Culinary Medicine**
80		Develop menus, including special diets	
81		Develop medically tailored meals	
82		Lifespan food and nutrition	Address **Culinary Nutrition** and **Culinary Medicine** across the lifespan and with populations, in altered eating and diet-related disease
83		Food and nutrition for healthy individuals	
84		Food and nutrition for diverse populations	
85		Altered eating conditions	
86		Diet-related diseases in individuals and populations	
87		Nutrition analysis - theoretical	Evaluate recipes, menus, food intake, and dietary patterns through theoretical and analytical nutrition analysis using evidence-based criteria to inform **Culinary Nutrition Activity**
88		Nutrition analysis - laboratory	
89		Nutrition assessment criteria	
90		Dietary analysis and modification	
91		Food and nutrition goal setting	

92	Domain 5: Culinary Nutrition: Food Science		
93	Domain 5: Culinary Nutrition: Food Science	Food and kitchen chemistry	Apply knowledge of food and kitchen chemistry, flavour science, and ingredient functionality to **Culinary Nutrition Activity**
94		Flavour science	
95		Ingredient functionality	
96		New product development	Conduct new product and recipe development through understanding of food, chemical and nutrient reactions, functional substitutions and food technologies in **Culinary Nutrition Activity**
97		Recipe development for product quality and performance	
98		Chemical reactions in food production, storage, preparation and cooking	
99		Nutrient/Non nutrient reactions in food and cooking	
100		Functional substitutions with food and cooking	
101		Current and future food technologies, and impact of nutrient content of foods	
102		Food safety theory	Apply food safety theory, preservation methods, and food and safety-related microbiology knowledge to **Culinary Nutrition Activity**
103		Food preservation methods	
104		Food and safety-related microbiological knowledge	
105		Multimodal sensory perception theory and practice	Incorporate insights from multimodal sensory perception, gastrophysics, and the science of pleasure, altered taste and compensatory strategies in **Culinary Nutrition Interventions**
106		Gastrophysics	
107		Science of pleasure	
108		Altered taste conditions	
109		Compensatory strategies in altered taste	
110		Sensory evaluation	Conduct sensory evaluation and testing of food and recipes using validated, rigorous methods in **Culinary Nutrition Activity**
111		Sensory testing of food using validated methods	
112		Sensory testing of recipes using validated methods	
113	Domain 6: Culinary Nutrition: Food Systems, Sustainability and Sovereignty		
114	Domain 6: Culinary Nutrition: Food Systems, Sustainability and Sovereignty	Food commodities, systems and sustainability	Understand sustainable food systems, supply chains, agriculture and farming, primary and secondary food production, food regulations and governance as applied to **Culinary Nutrition**
115		Food supply chains	
116		Agriculture and farming	
117		Primary food production	
118		Secondary food production	
119		Food regulations	
120		Food governance	
121		Ethical food and ingredient sourcing	Support ethical sourcing, sustainable procurement practices and work with farmers, producers and food artisans in **Culinary Nutrition Activity**
122		Sustainable procurement practices	
123		Farmer, producer and food artisan collaboration	
124		Food security	Advocate for food security, food justice and food sovereignty in **Culinary Nutrition Interventions**
125		Food justice	
126		Food sovereignty	
127		Advocacy in food security, justice and sovereignty	
128		Horticulture integrated culinary nutrition activity	Promote and provenance with horticulture integrated **Culinary Nutrition Activity** in kitchen and community gardens, sensory gardens, edible landscapes, urban farms and with **Produce Prescription Programs**
129		Provenance	
130		Kitchen gardens	
131		Community gardens	
132		Sensory gardens	
133		Edible landscapes	
134		Urban farms	
135		Produce prescription programs	
136		Food waste	Implement strategies to reduce food waste, support the circular economy, and promote sustainable diets in **Culinary Nutrition Activity**
137		Circular economy	
138		Sustainable diets and climate change	
139	Domain 7: Culinary Nutrition: Cultural Diversity		
140	Domain 7: Culinary Nutrition: Cultural Diversity	Cultural foodways	Embrace cultural foodways, 'traditional' diets, and recognise the psychosocial role of food in **Culinary Nutrition Activity**
141		Traditional diets	
142		Psychosocial role of food	
143		Research methods e.g. ethnography	Adopt methods in **Culinary Nutrition** research to explore and track historical and current dietary acculturation
144		Dietary acculturation	
145		Tracking dietary behaviours	
146		First Nations foodways	Respect First Nations foodways and Indigenous knowledges in **Culinary Nutrition Activity**
147		Indigenous knowledges	
148		Special dietary requirements for religions	Apply special dietary requirements for religious and cultural diets in **Culinary Nutrition Activity**
149		Special dietary requirements for cultural diets	
150		Cultural safety	Practice culturally safe care and self-reflection in Culinary Nutrition Interventions
151		Culturally safe care	
152		Self-reflection in culturally safe practice	

Domain 8: Culinary Nutrition: Communication and Media		
Domain 8: Culinary Nutrition: Communication and Media	Campaigns in culinary nutrition	Design and deliver impactful **Culinary Nutrition** campaigns with effective storytelling
	Effective storytelling	
	Social media strategies	Implement contemporary and emerging communication strategies for **Culinary Nutrition** translation
	Digital strategies e.g. live streamed	
	Emerging communication strategies	
	Culinary nutrition translation	
	Food trends	Track food, flavour, culinary and dining trends to inform targeted **Culinary Nutrition Interventions**
	Flavour trends	
	Culinary trends	
	Eating behaviour and dining trends	
	Food media	Craft and publish compelling **Culinary Nutrition** food media, proven recipes and multi-media communication
	Tested and proven recipe writing	
	Multi-media communication	
	Visual assets	Create impactful **Culinary Nutrition** visual assets through graphic design, food styling, photography and videography
	Graphic design	
	Food styling	
	Food photography	
	Food videography	

References

Croxford, S., Stirling, E., MacLaren, J., McWhorter, J. W., Frederick, L., & Thomas, O. W. (2024). Culinary Medicine or Culinary Nutrition? Defining Terms for Use in Education and Practice. Nutrients, 16(5), 603. *https://doi.org/10.3390/nu16050603

Mozaffarian, D., Blanck, H. M., Garfield, K. M., Wessing, A., & Petersen, R. (2022). A Food is Medicine approach to achieve nutrition security and improve health. Nature Medicine, 28(11), 2238–2240.
